# Sex-specific regulation of IL-10 production in human adipose tissue in obesity

**DOI:** 10.3389/fendo.2022.996954

**Published:** 2022-10-13

**Authors:** Narmadha Subramanian, Beatriz Tavira, Kaisa Hofwimmer, Beate Gutsmann, Lucas Massier, Julie Abildgaard, Anders Juul, Mikael Rydén, Peter Arner, Jurga Laurencikiene

**Affiliations:** ^1^ Lipid laboratory, Unit of Endocrinology, Department of Medicine Huddinge, Karolinska Institutet, Stockholm, Sweden; ^2^ Medical Department III – Endocrinology, Nephrology, Rheumatology, University of Leipzig Medical Center, Leipzig, Germany; ^3^ Centre for Physical Activity Research, Rigshospitalet, University of Copenhagen, Copenhagen, Denmark; ^4^ Danish Diabetes Academy, Odense University Hospital, Odense, Denmark; ^5^ Department of Growth and Reproduction, Copenhagen University Hospital-Rigshospitalet, Copenhagen, Denmark; ^6^ Department of Clinical Medicine, University of Copenhagen, Copenhagen, Denmark

**Keywords:** IL-10, sex-specific, adipose, macrophages, obesity, women, men

## Abstract

**Background:**

Obesity-associated metabolic complications display sexual dimorphism and can be impacted by cytokines. We previously showed that interleukin-10 (IL-10) was upregulated in white adipose tissue (WAT) of obese women with type 2 diabetes (T2D). Whether this pertains to men is unknown. The aim of this study was to compare the impact of obesity and T2D on WAT IL-10 levels in men versus women.

**Methods:**

Plasma and subcutaneous WAT biopsies were obtained from 108 metabolically well-characterized individuals. WAT *IL10* expression/secretion and WAT-resident IL-10-secreting macrophage number were measured. Circulating sex hormone levels were correlated to WAT *IL10* expression in 22 individuals and sex hormone effects on macrophage *IL10* expression were investigated *in vitro*.

**Results:**

Obese women with T2D showed increased *IL10* expression/secretion and IL-10-secreting WAT macrophage number compared to other female groups. This difference was absent in men. Non-obese women and men with T2D showed similar IL-10 levels compared to healthy controls, indicating that T2D alone does not regulate IL-10. Although WAT *IL10* expression correlated with serum estrone (E1) concentrations, recombinant E1 did not affect macrophage *IL10* expression *in vitro.*

**Conclusion:**

WAT IL-10 levels are higher in women with obesity and T2D, but not in men and this effect is primarily attributed to obesity *per se*. This is less likely to be driven by circulating sex hormones. We propose that the WAT IL-10 might exert protective effects in obesity-associated chronic inflammation in women which could be one of the contributing factors for the decreased morbidity observed in women during obesity than men.

## Introduction

Chronic low-grade inflammation in white adipose tissue (WAT) is important for the development of insulin resistance (IR) and type 2 diabetes (T2D) in obesity (1). This low-grade inflammation is characterized by the infiltration and phenotypic change of macrophages as well as other pro-inflammatory immune cell populations like CD8^+^T, dendritic and B cells (2–5). The infiltration of pro-inflammatory cells is associated with an increased production of chemokines and pro-inflammatory cytokines like CC-motif Chemokine Ligand 2 (CCL2), Tumor Necrosis Factor-α (TNFα), interleukins IL-1β and IL-6 (6–9). Anti-inflammatory cytokines like IL-10 are also regulated by obesity (10, 11).

It is evident that sex influences metabolic disorders like T2D and cardiovascular disease (CVD) (12). Body fat amounts and distribution differ between men and women, where men usually accumulate fat centrally and women peripherally (13). Females have about 10% higher total body fat content than males with the same body mass index (BMI) (14). However, during the reproductive years, women with obesity are better protected from T2D and CVD compared to men, which may at least in part be attributed to the inflammation/immune cell status in WAT (15, 16).

Inflammation has been shown previously to be dependent on age, sex, and ethnicity (17–19). Sex-specific differences are seen in levels of circulating inflammatory cytokines like IL-6, TNFα and also C-reactive protein (CRP) (20) as well as in both arms of immunity (adaptive and non-adaptive) (19, 21). Furthermore, antigen presentation and type I interferon response by dendritic cells are higher in women than in men whereas the number of natural killer cells, pro-inflammatory cytokine production and expression of toll-like receptors are higher in males compared to females (22–25). Thus, sex-specific effects on immune responses may contribute to different susceptibility to cardio-metabolic conditions between men and women.

Variations in inflammatory response between men and women could at least partially depend on sex hormones. Both testosterone (TS) and estrogen have immunomodulatory effects (26–28) where TS has immunosuppressive potency (26–28). Abdominal subcutaneous (sc) and visceral (vis) WAT aromatise androstendione and TS to estrone (E1) and estradiol (E2), respectively (29). Therefore, the local conversion of sex hormones might impact WAT metabolism and inflammation.

Previous studies suggest that IL-10 may have a specific role in obesity/T2D. Both circulating and WAT-expressed IL-10 are upregulated by obesity in women (10, 11, 30) and WAT IL-10 is strongly linked to IR (30). Furthermore, in WAT, pro-inflammatory macrophages (CD14^+^CD206^+^CD11c^+^ cells), that have been shown to correlate with IR in human WAT (31), are the main producers of IL-10 (30). In addition, *IL10* expression has been shown to be impacted by sex in different clinical contexts (17, 32, 33). Having in mind anti-inflammatory effect of IL-10 and better protection from obesity-associated metabolic complications in women compared to men, we aimed to compare the impact of obesity and T2D on WAT IL-10 levels in men versus women, intending to define the role of sex in IL-10 production under WAT metabolic challenge.

## Materials and methods

### Subjects

Cohort 1 consisted of non-obese (BMI<30) healthy, non-obese T2D and obese (BMI ≥30) T2D men and women. Clinical parameters of included individuals are shown in **Table 1**. T2D patients were mainly treated by metformin; two men (one non-obese and one obese) and two women (one non-obese and one obese) were also treated with insulin. The diabetes duration was similar for men (10 ± 8 years) and women (11 ± 9 years). Homeostatic model assessment of insulin resistance (HOMA-IR) was used to measure insulin sensitivity and was calculated from the levels of fasting glucose and insulin (34). Fat cell volume (pL) was measured as described previously (35). In brief, the diameter of 100 cells was measured by light microscopy using a scale on the objective. The average fat cell volume was calculated using the formula (π/6) X (3σ^2^xd + d^3^) (where d is the mean diameter and σ is the standard deviation of the diameter), which considers the fact that the cube of the diameter (d3) is skewed. Cohort 1 was utilized for the analysis of IL-10 and TS secretion in WAT explants, WAT IL10 mRNA expression, IL-10-secreting cells by flow cytometry, and IL-10 in plasma. Cohort 2 has been described previously in (36). In brief, clinical characteristics of the individuals of cohort 2 (51 obese women and 19 obese men with and without T2D) are shown in **Table 2**. IL-10 expression in visWAT and scWAT from these individuals was used for correlation analyses with HOMA-IR. Correlation analyses of IL10 mRNA expression with plasma sex hormone levels were performed in 22 women with obesity from cohort 3 (37). The clinical parameters of included individuals are listed in **Table 3**. In addition, stromal vascular fraction obtained from scWAT of four healthy female individuals undergoing plastic surgery were used for the analysis of IL-10 expression upon hormone stimulation in vitro. Only age and BMI from these individuals were available to us (age 48 ± 10 years, BMI 31 ± 5).

**Table 1:** Clinical parameters of cohort 1.

	Non-obese			Non-obese T2D			Obese T2D		
	Women	Men	p-value	Women	Men	p-value	Women	Men	p-value
Sample No	23	22		12	30		9	12	
Age	57 ± 7 (41 - 69)	59 ± 7 (43 - 69)	0.475	61 ± 4 (53 - 70)	*57 - 64 (41 - 69)	0.950	60 ± 5 (51 - 68)	60 ± 4 (52 - 66)	0.861
BMI *(kg/m^2^)*	25 ± 2 (22 - 29)	25 ± 2 (22 - 29)	0.652	26 ± 3 (23 - 30)	26 ± 2 (23 - 30)	0.999	33 ± 3 (30 - 40)	*31 - 35 (30 - 43)	0.986
Fat cell volume *(pL)*	*407 - 542 (210 - 1295)	542 ± 100 (368 - 770)	0.104	601 ± 123 (419 - 793)	551 ± 138 (336 - 900)	0.290	715 ± 128 (526 - 919)	773 ± 169 (531 -1062)	0.407
WHR	0.85 ± 0.05 (0.77 - 0.99)	0.95 ± 0.05 (0.84 - 1.04)	**<0.001**	*0.93 - 1 (0.87 - 1.2)	0.95 ± 0.04 (0.86 - 1.04)	0.853	0.97 ± 0.03 (0.9 - 1.02)	1.0 ± 0.04 (0.97 - 1.13)	**0.005**
TG *(mmol/L)*	1.02 ± 0.33 (0.45 - 1.8)	*0.73 - 1.42 (0.55 - 3.4)	0.438	*0.83 - 2.6 (0.3 - 6.8)	*0.77 - 1.6 (0.33 - 3.6)	0.436	1.16 ± 0.48 (0.44 - 2)	1.7 ± 0.73 (0.73 - 3.1)	**0.041**
Fasting insulin *(μIU/L)*	4.5 ± 1.2 (2.5 – 6.9)	*4.1 - 9.1 (2.4 - 14.2)	0.524	9.18 ± 6 (0.9 - 19.7)	*4.3 - 10.8 (2.1 - 24)	**0.007**	*5.2 - 18.3 (4.9 - 33.3)	*7 - 18.5 (1.1 - 67.1)	0.574
Fasting Glucose (*mmol/L*)	5.3 ± 0.3 (4.7 - 5.9)	*5.3 - 5.6 (5.1 - 6.7)	0.140	*7.1 - 9.3 (4.9 - 20.2)	7.4 ± 1.3 (5 - 10.8)	0.108	7.3 ± 0.6 (6.2 - 8.3)	*6.5 - 11.5 (6.2 - 13.6)	0.875
HbA1C *(mmol/mol)*	36 ± 3 (28 - 44)	34 ± 4 (24 - 44)	0.119	57 ± 15 (42 - 84)	47.6 ± 7.7 (34 - 65)	**0.006**	46 ± 5 (39 - 55)	55 ± 12 (43 - 76)	**0.037**
HOMA-IR	1.1 ± 0.3 (0.5 - 1.6)	1.6 ± 0.7 (0.5 - 3.2)	**0.007**	*1.1 – 4.5 (0.35 - 17.7)	*1.7 - 3.3 (0.5 - 7.4)	0.679	4.2. ± 3.2 (1.3 - 11.4)	*2.1 – 6.2 (0.67 - 40.6)	0.641

For normally distributed values, the mean ± SD with minimal and maximal values is shown. For non–normally distributed values (indicated with *), median–interquartile range with minimal and maximal values are indicated. Unpaired parametric (t-test) and non-parametric (Mann-Whitney)-tests were used to test the significance between men and women for normally and non-normally distributed (indicated with*) values, respectively. Significant values are indicated in bold.

**Table 2:** Clinical parameters of cohort 2.

	*Obese without T2D*			*Obese T2D*		
	*Women*	*Men*	*p-value*	*Women*	*Men*	*p-value*
Sample No	29	10		22	9	
Age *(years)*	45 ± 11 (25 - 63)	45 ± 12 (22 - 59)	0.817	*40 - 62 (31 - 65)	55 ± 10 (39 - 67)	0.401
BMI *(kg/m2)*	47 ± 5 (39 - 57)	47 ± 7 (37 - 58)	0.760	51 ± 7 (33 - 64)	46 ± 8 (38 - 58)	0.140
TG *(mmol/L)*	1.1 ± 0.4 (0.18 - 2.0)	*0.9 -1.6 (0.8-5)	0.273	*1.3 - 2.3 (0.6 - 11.2)	1.5 ± 0.6 (0.8 - 2.3)	0.395
Fasting insulin *(μIU/L)*	*11.1 - 27.6 (3.6 - 88.5)	15.1 ± 8.2 (3.8 - 30.2)	0.378	*15.1 - 46.8 (2.7 - 98.9)	*7.6 - 19.9 (2.2 - 111.3)	0.103
Fasting glucose*(mmol/L)*	*4.7 - 5.6 (4.1 - 10.4)	5.4 ± 0.8 (4.4 - 7.3)	0.606	7.4 ± 1.9 (4.5 - 12.1)	*5.6 - 7.1 (5.2 - 13.4)	0.287
HbA1C *(mmol/mol)*	35.1 ± 4.5 (27.9 - 47.7))	*35.5 - 39.4 (32.2 - 54.2)	0.072	*43.1 - 52.2 (34.5 - 86)	47 ± 10 (38 - 64.4)	0.402
HOMA-IR	*2.10 - 6.1 (0.64 - 18.4)	2.3 ± 1.4 (0.5 - 4.3)	0.073	*3.8 - 14 (0.6 - 30.5)	*3.7 - 6.8 (2.6 - 58.4)	0.321

For normally distributed values, the mean ± SD with minimal and maximal values is shown. For non–normally distributed values (indicated with *), median–interquartile range with minimal and maximal values is indicated. Unpaired parametric (t-test) and non-parametric (Mann-Whitney)-tests were used to test the significance between men and women for normally and non-normally distributed (indicated with*) values, respectively.

**Table 3:** Clinical parameters of cohort 3.

Factors	Values
Sample number	22
Age *(years)*	*32 - 41 (23 - 45)
BMI *(kg/m^2^)*	37 ± 4 (31 - 49)
WHR	0.94 ± 0.07 (1.1 - 0.78)
TG *(mmol/L)*	1.5 - 0.7 (3.1 - 0.4)

For normally distributed values, the mean ± SD with minimal and maximal values is shown. For non–normally distributed values (indicated with *), median–interquartile range with minimal and maximal values are indicated.

### Secretion of cytokines and sex hormones

A venous blood sample for each subject was obtained for routine clinical measurements, an abdominal scWAT biopsy was taken, and explant incubations were performed as previously described (35). IL-10 was quantified in adipose tissue explant incubation media and in serum samples using V-plex Plus human IL-10 detection kit (Mesoscale Discovery, K151QU, Research Boulevard, Rockville, US). 50 μl of each sample was analyzed according to manufacturer’s instructions, and data were collected with SECTOR Imager 6000 and analyzed with MSD Discovery Workbench software. Results are expressed in pg/ml. Estrone (E1) and estradiol (E2) concentrations were measured by LC-MS/MS technology as previously described in detail (38). Limits of detection for E1 and E2 were 2.9 pmol/L and 4.0 pmol/L, respectively. Serum concentrations of follicle stimulating hormone (FSH), leutinising hormone (LH) and sex hormone binding globulin (SHBG) were determined using a time-resolved fluoroimmunoassay (Delfia, Wallac, Turku, Finland). Detection limits for LH and FSH were 0.005 IU/L.

### Cell culture

Human monocyte cell line THP-1 (ATCC, Manassas, VA, USA) was cultured as recommended in ATCC protocols (39). 50 ng/ml PMA was added for 48 hours (Sigma-Aldrich, St.Louis, MO, USA P1585-1Mg) to differentiate monocytes to macrophages and then cells were treated with 50 ng/ml E1 (E-075, Sigma-Aldrich), 10 ng/ml TS (A8380, 5-alpha-Androstan-17beta-ol-3-one, Sigma-Aldrich) and 50ng/ml of E2 (E1024, Sigma-Aldrich) for 24 hours. The concentration was selected by titration (data not shown) and was comparable to the previously published *in vitro* studies (40). No toxic effects, measured as lactate dehydrogenase activity, were observed (data not shown). In parallel, cells were also treated with 20 ng/ml of TNFα (T6674, Sigma-Aldrich). Stromal vascular fraction (SVF) from human WAT was purified and frozen as previously described (35, 41). Frozen SVF was thawed, seeded at a density of 300,000 cells/well in 24-well plates and incubated for 24 hours in RPMI medium supplemented with 10% fetal calf serum (FCS; Gibco, Thermo Fisher Scientific, Waltham, MA, USA) and 2% penicillin-streptomycin (Gibco) at 37 °C followed by hormone stimulation as described for THP-1 cells.

### RNA expression analysis

RNeasy Lipid Tissue Mini Kit (Qiagen, Cat.No.74804, Hilden, Germany), NucleoSpin RNA kit (Macherey-Nagel, Duren, Germany) and RNeasy Micro kit (Qiagen) were used for RNA extraction of lipid tissue, THP-1 and SVF, respectively. Concentration and purity of RNA were measured using a Nanodrop ND-1000 Spectrophotometer (Thermo Fisher Scientific). cDNA synthesis for THP-1 and SVF was performed using iScript cDNA synthesis kit (Bio-rad, Hercules, CA) and Super Script III (ThermoFisher Scientific) was used to reverse transcribe RNA from lipid tissue. Quantitative RT-PCR for IL-10 was performed using commercial TaqMan probes (Hs00961622_m1) at 60 °C (Thermo Fisher Scientific) on CFX96 Touch™ qPCR Detection System (Bio-Rad). Microarray profiling of scWAT from cohort 3 was performed using Affymetrix Clariom D platforms and analyzed as described in (37).

### Intracellular staining and flow cytometry

Cryopreserved SVF was thawed, immediately washed with PBS/0.5% bovine serum albumin/2 mM EDTA buffer, filtered and seeded overnight in non-tissue culture treated 6-well plates in-order to avoid firm attachment of adherent cells to the plate. Brefeldin A (BD Biosciences, San Diego, CA, USA), a protein transport inhibitor, was used at 1:1000 for the last 3 hours of incubation to inhibit the release of cytokines. After two washes and filtering, cells were incubated with viability aqua dye for 30 minutes. The cells were then incubated with an antibody cocktail (42) for 30 minutes to stain for extracellular markers (BD Biosciences; Biolegend, San Diego, CA, USA; Thermo Fisher scientific). Research resource identifiers (RRIDs) of the antibodies are provided in the **Supplementary Table S1** (42). Subsequently, cells were fixed with 4% PFA at 4 °C for 20 minutes and stored at 4°C using freshly prepared PBS/1% FCS buffer. Next day the cells were permeabilized using BD Perm/wash™ buffer (BD Biosciences) and stained with IL-10 antibody for 45 minutes at 4°C (**Supplementary Figure S1**) (42). BD Fortessa equipped with 405 nm, 488 nm, 561 nm, and 640 nm lasers and with Diva software (BD Biosciences) was used to analyze all the samples. Fluorescence minus one (FMO) control was used for all the antibodies to set marker-positive cell populations. Data analysis was performed with FlowJo software (Tree Star, Ashland, OR, USA) where live/dead cells were first gated based on aqua staining, followed by gating for the single cells, and subsequently for monocytes/macrophages (CD45^+^CD14^+^). The M2 adipose tissue macrophages were defined as CD206^+^CD11c^-^ and M1 as CD206^+^CD11c^+^. Relative number of IL-10-secreting macrophages was then determined in the latter population.

### Statistical analysis

A statistical power calculation was made on available values for subcutaneous fat IL-10 secretion in 99 subjects including previously published cohort (n=80) (30) and additional cohort of 19 healthy individuals. According to calculations, 86 individuals were needed to detect 30% difference at alpha 0.05 and power 80%. Comparisons between the groups, correlations, linear regressions and graphical illustrations were done using GraphPad prism 7 (GraphPad Software, RRID: SCR_002798, La Jolla, CA). The Shapiro-Wilks test was used to determine the normal distribution of the variables, and logarithm transformation (log10) was performed for variables, such as IL-10, to achieve normal distribution. For normally distributed values, the mean ± SD with minimal and maximal values are indicated whereas median–interquartile range with minimal and maximal values are indicated for non–normally distributed values in the tables. Unpaired parametric (t-test) and non-parametric (Mann-Whitney)-tests were used to test the significance between two groups for normally and non-normally distributed values, respectively. Spearman correlation was used to evaluate association for non-normal distribution and Pearson correlation was used for all normally distributed factors. Data are presented as box plots in the graphs, showing minimum, first quartile, median, third quartile, and maximum values. Two-way ANOVA with Tukey’s and Sidak’s multiple comparisons were used in figures to compare metabolic groups of men and women and to compare the groups between men and women, respectively. A probability level of 0.05 was considered statistically significant.

## Results

### Participants

Clinical characteristics of cohort 1 are detailed in **Table 1**. To determine the effect of BMI and T2D separately, 108 patients from this cohort were classified into the following three groups: non-obese healthy (45 subjects), non-obese with T2D (42 subjects) and obese with T2D (21 subjects). Men and women included in the study were of similar age and matched for BMI in the respective subgroups. Furthermore, fat cell volume and plasma glucose were similar in men and women belonging to the same metabolic group. HOMA-IR and waist-to-hip ratio (WHR) were significantly higher in non-obese healthy men compared to women, whereas, fasting insulin and haemoglobin A1c (HbA1c) was higher in non-obese T2D women compared to men. In addition, WHR, triglycerides (TG) and HbA1c were significantly higher in men with obesity and T2D compared to women.

### IL-10 secretion and gene expression are regulated by obesity/T2D in women but not in men

**Figure 1:**
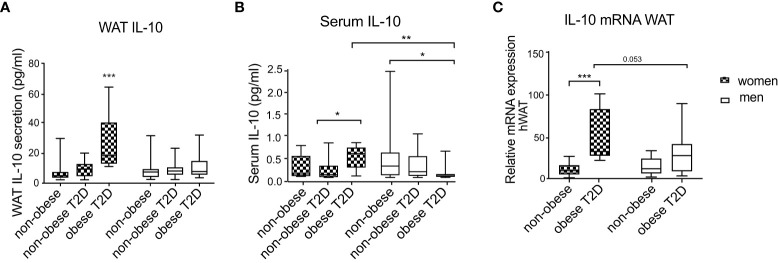
IL-10 secretion and mRNA expression in scWAT and serum of men and women. Three metabolic groups (non-obese healthy, non-obese T2D and obese T2D) of both women and men are represented by box plots. IL-10 secretion in WAT explants **(A)**, circulating serum IL-10 **(B)** and *IL10* mRNA expression in scWAT **(C)** are shown. Two-way ANOVA with Tukey’s and Sidak’s multiple comparisons were used in **(A–C)** to compare between the groups in men and women and to compare the groups between men and women, respectively. *Patients from cohort 1 have been used for this analysis and* number of individuals per group corresponds to **Table 1**, *** <0.001, ** <0.01, * <0.05.

To determine whether IL-10 levels are influenced by BMI, T2D and sex, we examined WAT IL-10 secretion in cohort 1. While IL-10 secretion was similar in healthy non-obese men and women, it was significantly increased in women with obesity and T2D compared to controls, but this increase was absent in men (**Figure 1A**). IL-10 release was also significantly higher in women with obesity and T2D compared to men from the same metabolic subgroup (**Figure 1A**). Non-obese women and men with T2D showed similar levels of WAT IL-10 secretion and no upregulation in comparison to healthy controls (**Figure 1A**), indicating that this cytokine is not regulated by T2D alone. Furthermore, in men, all three groups had similar levels of IL-10 secretion. To evaluate if this local sex-specific IL-10 regulation is also reflected in circulating levels, we determined IL-10 levels in serum of the same patient cohort. In serum of healthy non-obese men and women, IL-10 levels were similar (**Figure 1B**). Circulating IL-10 levels of healthy women did not differ from the other female groups. However, women with obesity and T2D had significantly higher circulating IL-10 compared to non-obese women with T2D (p=0.022) (**Figure 1B**). In contrast, IL-10 was lower in serum of men with obesity and T2D compared to non-obese healthy men (**Figure 1B**). Women with obesity and T2D had significantly higher circulating IL-10 compared to men belonging to the same metabolic subgroup (**Figure 1B**). Correlations of IL-10 to the inflammatory cytokines didn’t show sex differences where IL-10 was correlating positively with inflammatory factors in WAT and negatively in serum (**Supplementary Tables S2**, **S3** *).*

We further investigated if *IL10* levels are regulated transcriptionally (i.e., mRNA) level in WAT. Since non-obese T2D individuals showed similar IL-10 secretion compared to the control group, in this analysis we included only men and women from the healthy non-obese and obese T2D groups. The mRNA expression measurements revealed that *IL10* mRNA levels did not differ between healthy non-obese men and women. Similar to the secretion results, *IL10* expression was upregulated in women with obesity and T2D compared to healthy controls, and this regulation was not observed in men (**Figure 1C**). The difference between *IL10* expression in men and women with obesity and T2D had a borderline significance (p=0.053) (**Figure 1C**). Sex differences were further confirmed by positive correlations of WAT IL-10 production to the measures of glucose metabolism (plasma glucose, HOMA-IR and HbA1C) found in women, but not in men (**Supplementary Table S4**).

It is widely accepted that in obesity, inflammation is even more pronounced in visceral fat. To further investigate whether this sex-specific regulation of WAT IL-10 is also seen in visWAT, we used another cohort of obese individuals (cohort 2) where visWAT samples were available. Because all individuals in this cohort were obese, we could not evaluate effect of obesity and T2D due to the lack of the control group. Therefore, we evaluated if visWAT IL-10 levels were associated with metabolic status of these obese individuals measured by HOMA-IR. In visWAT, *IL10* did not correlate with HOMA-IR neither in men nor in women (**Supplementary Figure S2**), whereas scWAT IL-10 had a trend towards a positive correlation (p=0.096) in women and but not in men (**Supplementary Figure S2**) indicating that the sex specific regulation of IL-10 in obesity/T2D is only attributed to scWAT.

### Women with obesity and T2D have higher numbers of IL-10-positive macrophages than men with obesity and T2D

**Figure 2:**
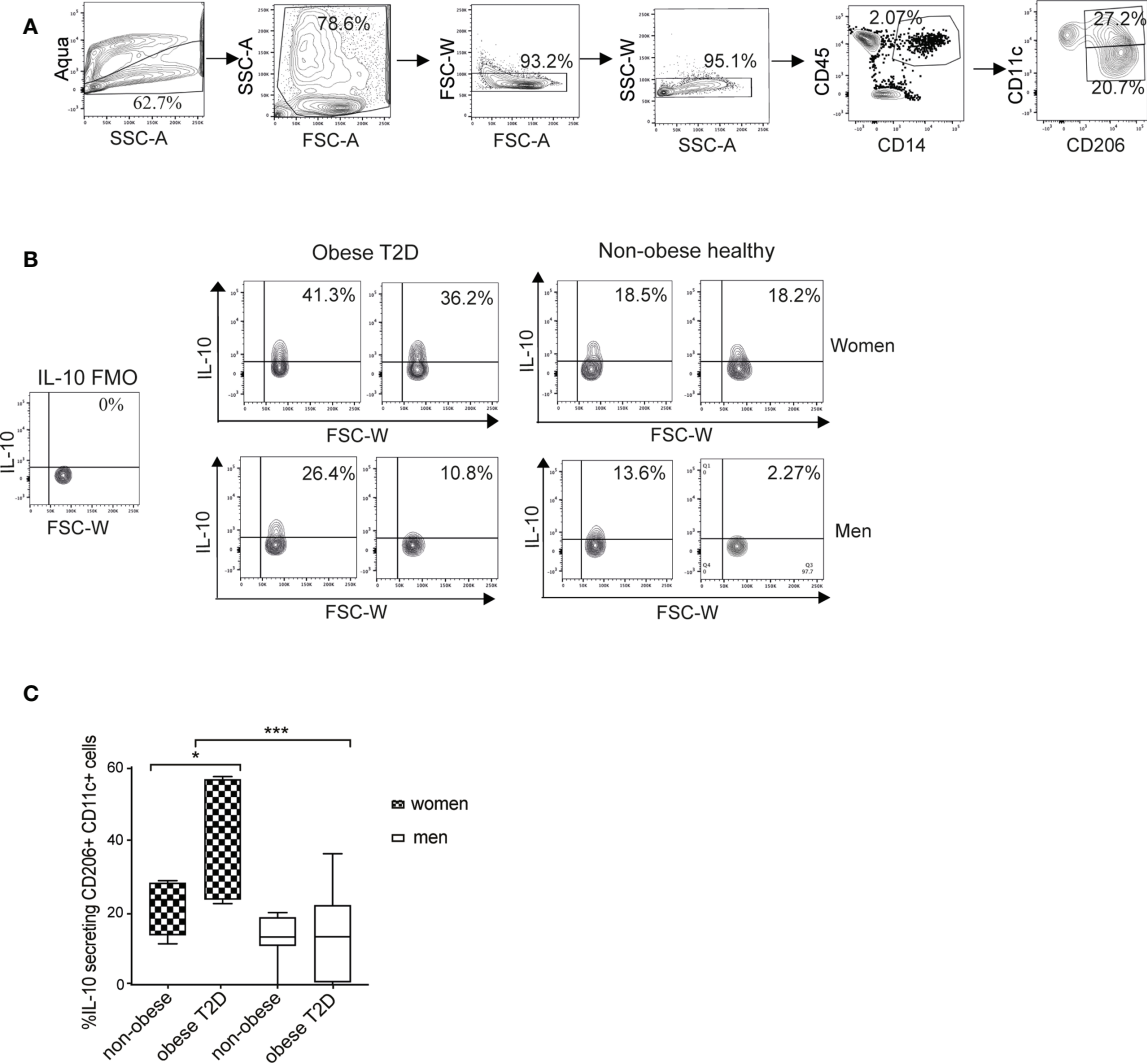
*Amount of IL-10 secreting macrophages is increased in scWAT of women with obesity and T2D but not in men.* **(A)** A flow cytometry gating strategy. Selection of alive single cells (four panels on the left), macrophage/monocyte (CD45^+^/CD14^+^) and M1 macrophage (CD45^+^/CD14^+^/CD206^+^/CD11c^+^) selection (two right-side panels). **(B)** The M1 macrophage population was then analyzed for IL-10 secretion. Representative contour plots from two individuals/group are shown for obese T2D and non-obese healthy women and men. FMO control samples are shown on the left side. **(C)** Compiled results of IL-10-secreting macrophage populations for non-obese healthy (n=8 for both men and women) and obese T2D (n=7 for women, n=8 for men) men and women *from cohort 1*. Two-way ANOVA with Tukey’s and Sidak’s multiple comparisons were used in **(C)** to compare between the groups in men and women and to compare the groups between men and women respectively. *** <0.001, * <0.05.

The M1 macrophages (CD11c+CD206+) are the major source of IL-10 in WAT (30). Therefore, we examined the possible difference in the amounts of IL-10-secreting cells between men and women. For this purpose, we established an intracellular staining protocol to detect cytokines in ex vivo WAT macrophages (**Supplementary Figure S1**). To quantify IL-10-expressing cells, we used a gating strategy detailed in **Figure 2A** and in the material and methods. Data analysis visualized by representative images of the four groups (**Figure 2B**) and the compiled results (**Figure 2C**) demonstrated that healthy women and men showed no difference in the IL-10-secreting cell fraction while women with obesity and T2D had significantly higher proportion of IL-10-secreting macrophages compared to all other examined groups (**Figure 2C**). In men, the amounts of IL-10-secreting cells were not different in the healthy non-obese compared to individuals with obesity and T2D.

### *IL10* expression correlates with estrone secretion

We next examined if the sex-specific differences in IL-10 regulation by obesity and T2D associated with the levels of circulating hormones linked to the gonadotropin axis (FSH, LH, SHBG, E1 and E2). For this, we used available samples from cohort 3 (37). We found weak but significant correlations of E1 (r=0.221 and p=0.027) and free E1 (r=0.216 and p=0.029) with WAT IL10 expression (**Table 4**). All the patients in this cohort were obese and there was no correlation between IL-10 and BMI (p=0.139). Therefore, the IL-10/E1 correlation was not driven by BMI in this cohort.

**Table 4:** Correlations of serum hormones with human WAT IL10 mRNA expression in cohort 3 (n=22).

	Linear regression	
Hormones	r-value	p-value
E1	**0.221**	**0.027**
E2	0.053	0.303
FSH	< 0.001	0.923
LH	0.142	0.084
SHBG	0.034	0.413
Free E1	**0.216**	**0.029**
Free E2	0.059	0.275

p- and r- values of Pearson correlations are indicated. Significant values are indicated in bold.

### Recombinant E1 does not upregulate IL10 expression *in vitro*

**Figure 3:**
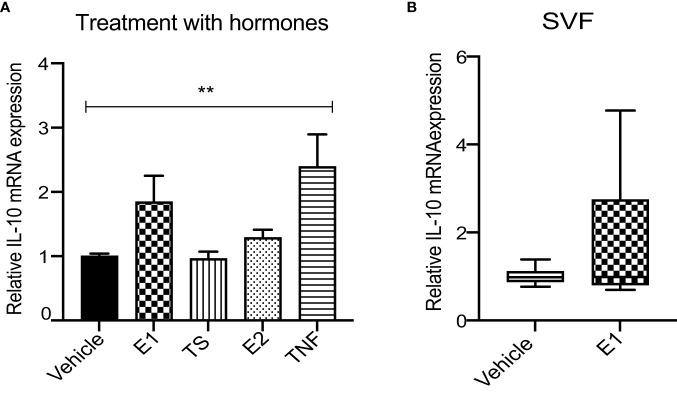
*E1 has no effect on IL-10 expression neither in in vitro stimulated THP-1 macrophages nor in primary WAT SVF.* **(A)** Relative *IL10* mRNA expression in E1, TS, E2 and TNFα-treated THP-1 macrophages. Two-way ANOVA with Sidak’s multiple comparison was used to test the significance. ** <0.01 (n=4). **(B)** Relative *IL10* mRNA in E1-stimulated WAT SVF; (Mann-whitney test, n=4 subjects in triplicates).

Finally, to investigate whether the observed correlation between circulating E1 and WAT IL10 expression could reflect causality, we examined the effect of recombinant human E1 (rhE1) on IL10 expression *in vitro*. Differentiated THP-1 cells were stimulated by rhE1, rhE2, TS or TNFα (as a positive control) and IL10 expression was measured. The results demonstrated no significant effect of rhE1 or any other tested hormone on IL10 mRNA levels (**Figure 3A**). In addition, no effect of rhE1 was observed on primary WAT SVF cells (**Figure 3B**).

## Discussion

In this study, we demonstrate that although WAT IL-10 levels are similar in non-obese men and women, T2D in combination with obesity is linked to a marked increase in IL-10 in scWAT of women, but not men. Furthermore, numbers of IL-10-secreting macrophages are increased in WAT of women with obesity and T2D, but not in men. Together, the findings suggest a sex-specific regulation of this cytokine upon WAT mass expansion. In contrast, circulating IL-10 levels were mostly affected (reduced) in men with obesity and T2D. This indicates that obesity/T2D has different impacts on circulating and WAT IL-10. Although WAT IL10 mRNA significantly correlates with circulating E1, we could not establish a direct effect of this hormone on IL10 expression using *in vitro* cell models.

As discussed earlier, sexual dimorphism in regulation of pro- and anti-inflammatory cytokines is observed in humans and animal models (20, 43–45). For example, female mice show higher periodontal bone loss than male mice, which is associated with higher inflammatory cytokines (IL-6, IL-1b and IL-17a) production (44). On the other hand, young female mice are protected against osteoarthritis and bone degeneration, which associates with higher levels of TGFb1 and IL-4 (45). It is also widely accepted that the pathogen (human immune deficiency virus, coxsachievirus, respiratory virus etc)-induced inflammation is sex dependent in both mouse and humans where males are more susceptible than females (46–48).

IL-10 has been shown to have a gender-specific regulation by several earlier studies not related to adipose tissue. IL-10 is significantly higher upregulated in men compared to women when exposed to endotoxin (17). Depression-related symptoms were shown to be associated with low circulating IL-10 levels in women but not in men (32). In addition, female IL-10 knockout mice are more prone to Pseudomonas aerigonas infection than male (33). Therefore, it is likely that IL-10 production is associated with sex-specific factors that are at present unknown.

Although sex hormones are mostly produced by ovaries and testis, they can also be aromatized in peripheral tissues like adipose, breast and brain tissues (49–51). Furthermore, their influence on cytokine and chemokine secretion is established (52, 53). For example, *in vivo* treatment of mice with estradiol has been shown to increase insulin sensitivity and decrease basal lipolysis in WAT, which was connected to lower levels of TNFα and IL-6 in serum (43). In this study, correlation analysis of sex hormones and WAT IL10 expression showed that only E1 was associated with increased IL-10 levels. Adipose tissue is a major site of aromatization of E1, the main circulating estrogen form in post-menopausal women (29). Furthermore, the production of circulating E1 is increased in obesity due to increased aromatization in adipose tissue (54). E1 treatment significantly increases IL-10 secretion in T cells (40) and increased circulating IL-10 levels are observed in post-menopausal compared to pre-menopausal women (55). Although we did not observe a direct effect of E1 or any other tested sex hormone on IL10 expression in WAT SVF or THP-1 macrophages *in vitro*, we cannot exclude that sex hormones (and E1 in particular) are playing a role in obesity-related IL-10 upregulation in women *in vivo*.

Our results suggest that sex-specific IL-10 regulation by obesity/T2D is mediated at the transcriptional level because mRNA expression was found to be increased in parallel to secretion. This induction occurs in the M1-like macrophage population in scWAT, but only in women with obesity and T2D. However, at present we do not know the molecular mechanism of this upregulation and which factor increasing IL-10 production is absent in men with obesity and T2D. Therefore, additional studies in larger cohorts and WAT-derived macrophages obtained from men are required. On the other hand, such cohorts with large adipose tissue samples obtained from men are difficult to access. For the same reason, it is not feasible to study possible sex differences for WAT IL-10 in lean (BMI<25) patients with T2D. For ethical reasons, visceral depot cannot be investigated in a clinical setting like cohort 1. We have previously shown that IL-10 production is positively correlated to HOMA-IR in a cohort of women with broad range of BMI. Even in a cohort of obese only women (cohort 2 in this study), IL-10 had a clear tendency for correlation to HOMA-IR. However, such association was completely absent in visceral fat of the same women cohort and in men (both scWAT and visWAT). Therefore, we suggest that IL-10 regulation in obesity is specific to female scWAT.

Several measures of glucose metabolism were found to be different in men versus women in specific metabolic groups. For example, heathy men had significantly higher HOMA-IR than women and obese T2D men had higher HbA1C than women in the same metabolic group. On the other hand, non-obese T2D women had higher insulin and HbA1C compared to men. Therefore, we cannot fully exclude those differences in glucose metabolism in itself could have affected IL-10 regulation presented in **Figure 1**. The fact that measures of glucose metabolism were not matched between sexes, can be considered as a limitation of this study.

Although the associations of IL-10 with obesity and metabolic complications have been studied previously (10, 30, 56–58), the regulation of IL-10 in different sexes has, to the best of our knowledge, not been addressed. Few IL-10 studies that included both genders (adolescent and young men and women) (57, 58) did not specifically examine the gender effect on IL-10 associations with metabolic abnormalities. We show that WAT and serum IL-10 levels of healthy men and women are the same, but a gender-specific effect is observed in connection to obesity. In murine models, Gotoh et al., demonstrated that high fat diet-induced obesity decreased serum IL-10 levels in male mice (59), which is similar to our current finding in men with obesity and T2D. The same study also suggested that the majority of IL-10 produced in the body is coming from spleen and this may be one of the reasons that the WAT-specific upregulation of IL-10 observed in women with obesity and T2D was not observed in circulation (59–61).

In summary, there is a sex-specific obesity/T2D regulation of the anti-inflammatory cytokine IL-10 in scWAT and in serum. We propose that the scWAT IL-10 might exert protective effects in obesity-associated chronic inflammation but that this mechanism is absent in men. However, the molecular mechanisms for such sex-specific regulation remain to be revealed.

## Data availability statement

The original contributions presented in the study are included in the article/**Supplementary Material**. Further inquiries can be directed to the corresponding authors.

## Ethics statement

The studies involving human participants were reviewed and approved by Regional ethics board of the Stockholm County Council. The patients/participants provided their written informed consent to participate in this study.

## Author contributions

NS, BT, PA and JL designed a study, NS, BT, KH, BG, LM, JA, AJ, MR performed data collection and analysis, as well as participated in discussions of the results, NS and JL drafted the manuscript. All authors contributed to the article and approved the submitted version.

## Funding

Swedish research council (2016-00694), Novo Nordisk Foundation including NNF210C0069989, the Tripartite Immuno-metabolism Consortium (TrIC) Grant Number NNF15CC0018486 and the MSAM consortium NNF15SA0018346 and the Strategic Research Programme in Diabetes at Karolinska Institutet, KID grant at Karolinska Institutet.

## Acknowledgments

The technical assistance of Gaby Åström, Elisabeth Dungner, Kerstin Wåhlén, Yvonne Widlund and Katarina Hertel (Dept. of Medicine Huddinge, Karolinska Institutet, Sweden) is greatly appreciated. Cell sorting was performed at MedH Flow Cytometry Core Facility.

## Conflict of interest

The authors declare that the research was conducted in the absence of any commercial or financial relationships that could be construed as a potential conflict of interest.

## Publisher’s note

All claims expressed in this article are solely those of the authors and do not necessarily represent those of their affiliated organizations, or those of the publisher, the editors and the reviewers. Any product that may be evaluated in this article, or claim that may be made by its manufacturer, is not guaranteed or endorsed by the publisher.
