# Supplementary material for: Sex-specific regulation of IL-10 production in human adipose tissue in obesity
Source: Front Endocrinol (Lausanne). 2022 Oct 13;13:996954. doi: 10.3389/fendo.2022.996954 (PMC9606404; doi:10.3389/fendo.2022.996954)
Supplement: Supplementary file 1 [file DataSheet_1.pdf]

Suplementary Tables:

Table S1: Research resource identifiers (RRIDs) of the antibodies.

Marker	Conjugate	Clone	Source	Catalogue no.	Dilution	RRID
CD45	AF700	HI30	BD	560566	1:80	AB_1645452
CD14	BV711	MΦP9	BD	563373	1:80	AB_2744290
CD11c	Pe-Cy7	B-Iy6	BD	561356	1:80	AB_10611859
CD206	BV421	19.2	BD	564062	1:80	AB_2738570
CD163	BV605	GHI/61	BD	745091	1:40	AB_2742705
IL-10	APC	JES3-9D7	Biolegend	501410	1:20	AB_315176
Aqua dead cell stain			Invitrogen	L34957	1:1000	

Antibodies, clone, catalogue number, dilution for all the markers used for flow cytometry are listed along with RRIDs.

Table S2: Multiple regression of WAT IL-10 with other WAT cytokines

**IL-10**	**Women**		**Men**	
	**β-coeff**	**p-value**	**β-coeff**	**p-value**
IL-6	0.391	**0.01**	0.226	**0.033**
MCP-1	0.588	**<0.001**	0.349	**0.001**
TNFa	0.428	**0.006**	0.315	**0.003**

The analysis was performed in cohort 1 (n = 42 for women and n = 63 for men). Multiple regression analysis was performed to identify significant correlations of WAT IL-10 with three other WAT cytokines when corrected to BMI.

Table S3: Linear regression of serum IL-10 with other circulating cytokines

	Women (IL-10)		Men (IL-10)	
	r-value	p-value	r-value	P-value
IFNg	0.651	**<0.001**	-0.519	**<0.001**
IL-1b	-0.611	**0.001**	-0.491	**0.001**
IL-6	-0.679	**<0.001**	-0.622	**<0.001**
MCP-1	-0.622	**<0.001**	-0.618	**<0.001**
TNFa	-0.465	**0.001**	-0.678	**<0.001**
IL-1RA	-0.580	**<0.0001**	-0.639	**<0.001**

The analysis was performed in cohort 1 (n = 42 for women and n = 63 for men). Circulating IL-10 levels did not correlate to BMI. Therefore, linear regressions were used for association analysis. Pearson r- and p- values are shown (significant values in bold).

Table S4: Spearmen correlations of IL-10 production with measures of glucose metabolism

**Women**	**p-glucose**		**HbA1c**		**HOMA-IR**	
	**rho**	**p-value**	**rho**	**p-value**	**rho**	**p-value**
IL-10 WAT secretion	**0.286**	**0.066**	**0.378**	**0.013**	**0.414**	**0.006**
IL-10 mRNA	**0.736**	**<0.0001**	**0.676**	**0.001**	**0.742**	**<0.0001**
IL-10 Serum levels	-0.029	0.849	-0.193	0.203	0.086	0.569

**Men**	**p-glucose**		**HbA1c**		**HOMA-IR**	
	**rho**	**p-value**	**rho**	**p-value**	**rho**	**p-value**
IL-10 WAT secretion	0.156	0.221	0.124	0.331	0.170	0.186
IL-10 mRNA	0.270	0.235	0.097	0.682	0.431	0.057
IL-10 Serum levels	-0.025	0.839	-0.009	0.936	0.015	0.900

Spearman correlations were performed in cohort 1 (n = 42 for women and n = 63 for men).

Supplementary figures

Figure S1: Intracellular staining protocol for flow cytometry

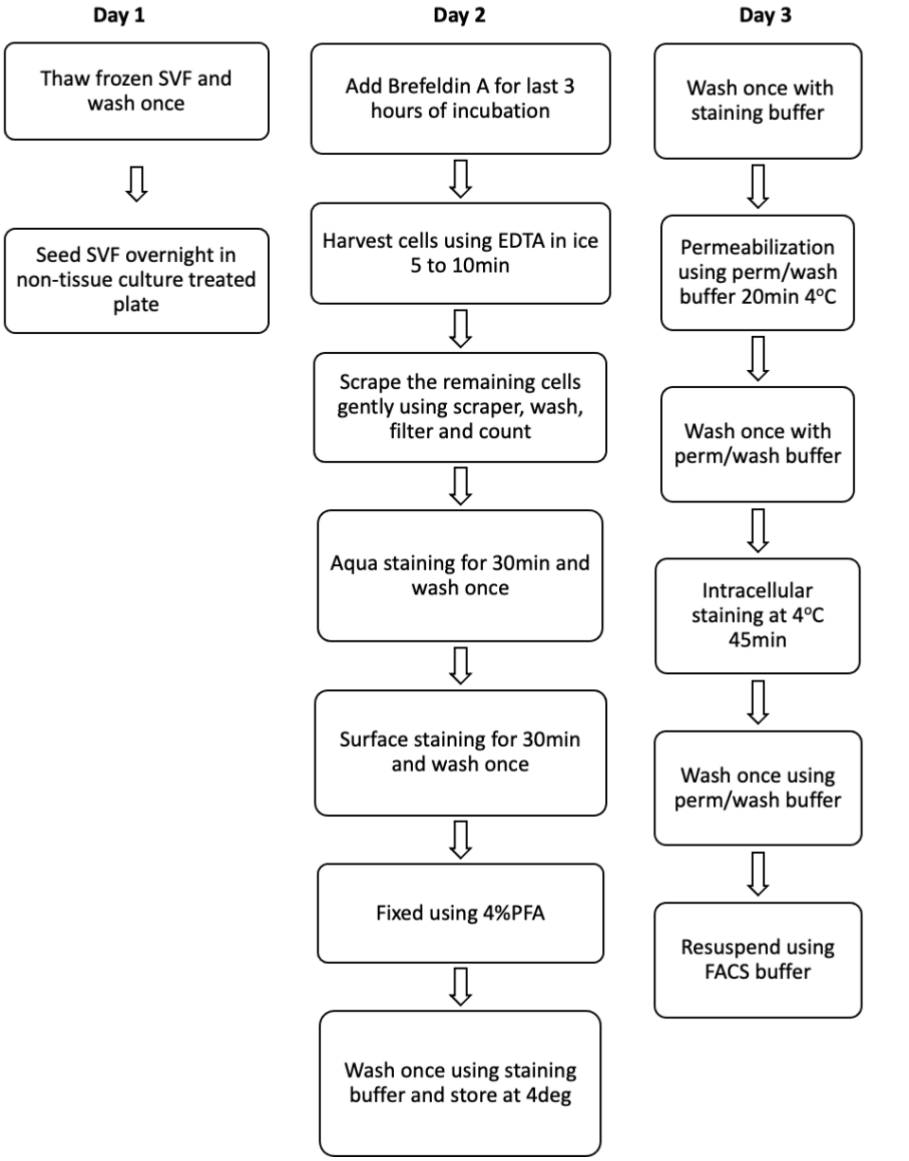

Step by step procedure for intracellular staining for flow cytometry is shown for day 1, 2 and 3.

Figure S2: Correlation of *IL10* to HOMA-IR in men and women in different depots

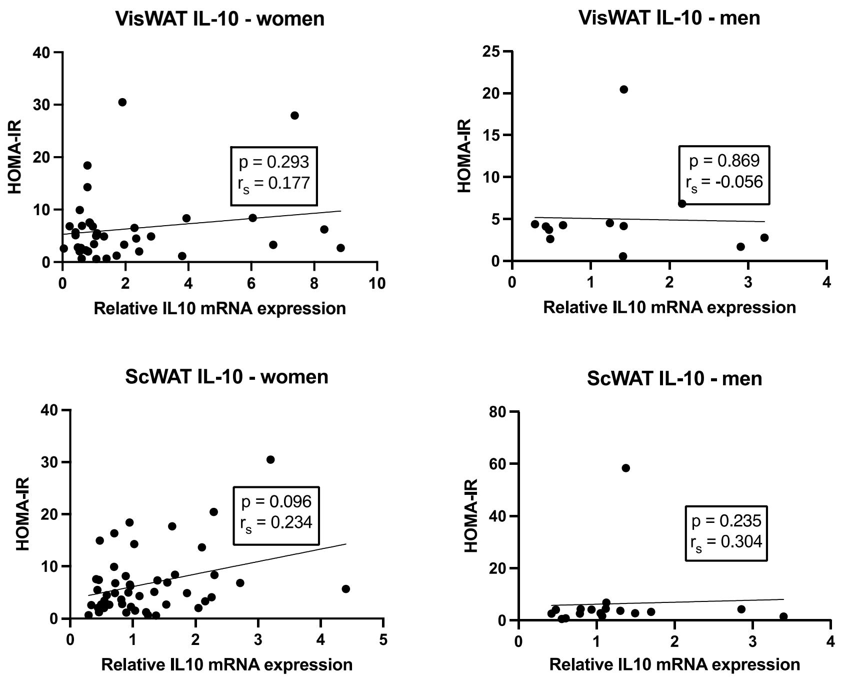

IL10 expression in scWAT and visWAT was plotted against HOMA-IR in both men and women from cohort 2 (scWAT n = 51 obese women and 18 obese men; visWAT n = 39 obese women and 15 obese men), Spearman correlation was used to evaluate association for not normally distribution.
